# Supplementary material for: Hybrid Models and Biological Model Reduction with PyDSTool
Source: PLoS Comput Biol. 2012 Aug 9;8(8):e1002628. doi: 10.1371/journal.pcbi.1002628 (PMC3415397; doi:10.1371/journal.pcbi.1002628)
Supplement: Text S4 — Complete source code for the PyDSTool package (version 0.88.120504). Includes API documentation and help files linking to web pages. This file is identical to the current public release on Sourceforge.net. (ZIP) [file pcbi.1002628.s004.zip › PyDSTool/ReadMe.html]

PyDSTool v0.88


# PyDSTool v0.88

May 2012. *This is a beta release version.*

---

  

### Getting started and documentation

See the online documentation,
particularly the GettingStarted and Tutorials pages! Please report
bugs and suggestions using the user forum linked to there.

Full API documentation can be found locally here. It is auto-generated from the
source code using Epydoc and is meant as a reference for functions and
classes only.

### Tests and examples

Tests and examples can be found in the /tests/ directory. Some tests can only be run once others have
produced their output, for instance HH\_loaded.py, HH\_loaded\_dopri.py, imp\_load\_test.py. Several of the examples
require an external compiler. An easy way to run all the tests in an appopriate order is to run the script
'run\_all\_tests.py' in the /tests/ directory. There is a simple option in that file that you may need to
edit in order to select whether the external compiler tests should be run (in case you do not have gcc
working on your system). Note that on some platforms you will see an error report when the script tries to
automatically close matplotlib graph windows, to the effect of:
"Fatal Python error: PyEval\_RestoreThread: NULL tstate".
This error can be ignored. You may also have to close the plot windows yourself before the script can continue.
This will depend on your platform and settings.

Source files in the installation root directory also contain basic tests of their own functions. Just
execute the files as if they were scripts.

### Change histories

Version change histories appear in the bzr
repository browser, and some older information is in the headers of each source file. An overview of the changes
in a new release can be found in the SourceForge release notes.

  

---

  

*## Credits:*

Coding and design by Robert Clewley, Erik Sherwood, Drew LaMar, and John Guckenheimer, except
where otherwise stated in the code or documentation. (Several other
open source codes have been redistributed here under the compatible licenses.)
